# (Pro)renin Receptor Triggers Distinct Angiotensin II-Independent Extracellular Matrix Remodeling and Deterioration of Cardiac Function

**DOI:** 10.1371/journal.pone.0041404

**Published:** 2012-07-23

**Authors:** Anne-Mari Moilanen, Jaana Rysä, Raisa Serpi, Erja Mustonen, Zoltán Szabò, Jani Aro, Juha Näpänkangas, Olli Tenhunen, Meeri Sutinen, Tuula Salo, Heikki Ruskoaho

**Affiliations:** 1 Department of Pharmacology and Toxicology, Institute of Biomedicine, University of Oulu, Oulu, Finland; 2 Department of Pathology, The Institute of Diagnostics, University of Oulu, Oulu, Finland; 3 Department of Diagnostics and Oral Medicine, Institute of Dentistry, Oulu University Hospital University of Oulu, Oulu, Finland; 4 Institute of Dentistry, University of Helsinki, Finland; University of Western Ontario, Canada

## Abstract

**Background:**

Activation of the renin-angiotensin-system (RAS) plays a key pathophysiological role in heart failure in patients with hypertension and myocardial infarction. However, the function of (pro)renin receptor ((P)RR) is not yet solved. We determined here the direct functional and structural effects of (P)RR in the heart.

**Methodology/Principal Findings:**

(P)RR was overexpressed by using adenovirus-mediated gene delivery in normal adult rat hearts up to 2 weeks. (P)RR gene delivery into the anterior wall of the left ventricle decreased ejection fraction (*P*<0.01), fractional shortening (*P*<0.01), and intraventricular septum diastolic and systolic thickness, associated with approximately 2–fold increase in left ventricular (P)RR protein levels at 2 weeks. To test whether the worsening of cardiac function and structure by (P)RR gene overexpression was mediated by angiotensin II (Ang II), we infused an AT_1_ receptor blocker losartan via osmotic minipumps. Remarkably, cardiac function deteriorated in losartan-treated (P)RR overexpressing animals as well. Intramyocardial (P)RR gene delivery also resulted in Ang II-independent activation of extracellular-signal-regulated kinase1/2 phosphorylation and myocardial fibrosis, and the expression of transforming growth factor-β1 and connective tissue growth factor genes. In contrast, activation of heat shock protein 27 phosphorylation and apoptotic cell death by (P)RR gene delivery was Ang II-dependent. Finally, (P)RR overexpression significantly increased direct protein–protein interaction between (P)RR and promyelocytic zinc-finger protein.

**Conclusions/Significance:**

These results indicate for the first time that (P)RR triggers distinct Ang II-independent myocardial fibrosis and deterioration of cardiac function in normal adult heart and identify (P)RR as a novel therapeutic target to optimize RAS blockade in failing hearts.

## Introduction

The key pathophysiological process that ultimately leads to chronic heart failure is myocardial remodeling in response to pressure overload due to hypertension or loss of muscle mass due to myocardial infarction [1]. Central features of myocardial remodeling are cardiomyocyte hypertrophy, apoptosis, fibrosis, impaired vascularization and changes in contractile function [1]. The renin-angiotensin-system (RAS) plays a pivotal role both in remodeling process and pathogenesis of heart failure. By interacting with angiotensin II (Ang II) subtype 1 receptors (AT_1_ receptors), Ang II induces cardiomyocyte hypertrophy, apoptosis and excessive myocardial fibrosis in hypertension induced left ventricular (LV) remodeling and post-infarction [2]. However, the precise function of recently discovered member of RAS, (pro)renin receptor ((P)RR), has not yet been solved. (P)RR is a receptor for renin and prorenin that on ligand binding not only increases local production of Ang I from angiotensinogen, but also induces Ang II-independent intracellular signaling [3].

To date the experimental evidence for the pathophysiological role of (P)RR is based on the phenotype analysis of transgenic animals overexpressing ubiquitously prorenin or (P)RR, and the use of a putative (P)RR blocker [4]–[7]. In rats overexpressing the (P)RR gene in vascular smooth muscle cells, (P)RR has resulted in elevated blood pressure, a rise in plasma aldosterone levels and increased renal cyclooxygenase-2 expression [4]. Moreover, rats overexpressing the (P)RR gene developed glomerulosclerosis with increased extracellular signal-regulated kinase 1/2 (ERK1/2) phosphorylation and transforming growth factor-β1 (TGFβ1) expression [5], [6]. Very recent studies have associated (P)RR with vacuolar-type (H^+^)-ATPase (V-ATPase) and the Wnt signaling during embryonic development [8], [9]. Cardiomyocyte-specific deletion of (P)RR resulted in heart failure and the mice died within 3 weeks of birth, the phenotypes observed being ascribed to V-ATPase loss of function [10]. Because overexpression of (P)RR targeted to the heart or cardiac-specific inducible (P)RR knockout has not been generated, the precise role of (P)RR in the adult heart remains unclear.

In the present study, we examined the direct myocardial effects of (P)RR on cardiac function. (P)RR was overexpressed by using adenovirus mediated gene delivery in normal rat hearts. Because these experiments revealed that local (P)RR gene delivery deteriorates LV systolic function, we evaluated several potential mechanisms triggering the myocardial remodeling by (P)RR gene overexpression. Furthermore, we infused an AT_1_ receptor blocker losartan to test whether the worsening of cardiac function by (P)RR gene overexpression is mediated by Ang II.

## Methods

### Ethics Statement

All experimental protocols were approved by the Animal Use and Care Committee of the University of Oulu and conform to the Guide for the Care and Use of Laboratory Animals Published by the US National Institutes of Health. Approval ID: ESHL-2008-01313/Ym-23, delivered the 06/03/2008.

### Recombinant Adenoviral Vectors

The (P)RR-overexpressing adenovirus (serotype 5) was generated by subcloning a full length coding region of rat (P)RR cDNA into *Sal*I and *Hind*III sites of the pShuttle-CMV vector (Qbiogene Inc., Illkirch, Cedex-France). The sequences for the cloning primers used were as follows; (P)RR forward 5′-GCG TCG ACC GTG GCA CCA TGG CTG TGC-3′ and reverse 5′-CCC AAG CTT TCA ATC CAT TCG AAT CTT CTG GTT TG-3′. The pShuttle-CMV contains CMV promoter and the SV40 polyadenylation signal. The pShuttle-CMV-LacZ was a commercial plasmid (Stratagene). (P)RR-pShuttle-CMV-vector was transformed into BJ5183-AD-1 cells (Stratagene, La Jolla, CA, USA) by electroporation. Vectors were transfected into AD-293A cells (Qbiogene Inc.) with Lipofectamine 2000 (Invitrogen). Adenoviruses were purified by centrifugation on iodixanol by standard methods (OptiPrep, Axis-Shield PoC AS, Oslo, Norway). The adenoviral titers were determined by AdEasy Viral Titer Kit (Stratagene) and β-galactosidase concentration by luminescent β-gal Kit (Clontech Laboratories Inc).

### Intramyocardial Gene Transfer and Losartan Treatment with Osmotic Minipumps

We and others have previously shown that local injection of adenoviral constructs into the LV free wall is an efficient site-specific method of gene delivery that targets high expression of the transgene in the left ventricle without affecting other organs or other regions of the heart and has a global effect of cardiac function [11], [12]. Male 8-week-old Sprague-Dawley rats weighing 250–300 g were anesthetized with medetomidine hydrochloride (Domitor, 250 µg/kg i.p.) and ketamine hydrochloride (Ketamine, 50 mg/kg i.p.). Rats were connected to the respirator through a tracheotomy. A left thoracotomy and pericardial incision was performed. Adenovirus-mediated gene transfer into the LV free wall was performed as previously described [13]–[15]. Different doses of adenoviral constructs were first tested to increase LV (P)RR mRNA levels at 2 weeks closely to those observed in experimental models of stroke-prone spontaneously hypertensive rat hearts [16] and with congestive heart failure due to coronary ligation [17]. Two weeks following (P)RR gene delivery protein levels were quantitatively equal to the (P)RR protein levels (about 2-fold higher compared to controls) observed in a post-MI rat hearts [18] and in dilated cardiomyopathy patients [18], when recombinant adenovirus (1×10^9^ infectious units, ifu), in a 100 µl volume, was injected using a Hamilton precision syringe directly into the anterior wall of the left ventricle. The syringe was inserted in one site of the LV free wall (apex to base), and then slowly the solution was injected while withdrawing the syringe. After the operation, anaesthesia was partially antagonized with atipamezole hydrochloride (Antisedan, 1.5 mg/kg i.p.) and rats were hydrated with physiological saline solution (5 ml s.c.). For postoperative analgesia, buprenorphine hydrochloride (Temgesic, 0.05–0.2 mg/kg s.c.) was administered.

Losartan (400 µg/kg/h) was administered via subcutaneously implanted osmotic minipumps (Alzet model 2001 for 1 week and Alzet model 2002 for 2 weeks; Scanbur BK AB, Sollentuna, Sweden), as described previously [19], [20]. Minipumps were implanted subcutaneously before the gene delivery. The dose of losartan used in the present study abolishes completely Ang II-induced increase in mean arterial pressure, LV weight/body weight ratio and elevation of skeletal α-actin, β-myosin heavy chain, atrial natriuretic peptide (ANP) and B-type natriuretic peptide (BNP) mRNA levels [20]. The total number of animals used was 161.

### Echocardiographic Measurements

Transthoracic echocardiography was performed using the Acuson Ultrasound System (Sequoia™ 512) and a 15-MHz linear transducer (15L8) (Acuson, MountainView, CA, USA) as previously described [13]–[15]. Before examination, rats were sedated with ketamine (50 mg/kg i.p.) and xylazine (10 mg/kg i.p.). Using two-dimensional imaging, a short axis view of the left ventricle at the level of the papillary muscles was obtained, and a two dimensionally guided M-mode recording through the anterior and posterior walls of the LV was obtained. LV end-systolic and end-diastolic dimensions as well as the thickness of the interventricular septum and posterior wall were measured from the M-mode tracings. LV fractional shortening (FS) and ejection fraction (EF) were calculated from the M-mode LV dimensions using the following equations:
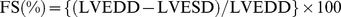


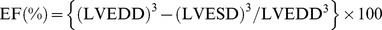



An average of three measurements of each variable was used. All echocardiographical measurements were performed by skilled sonographers (E.M. and Z.Sz.) blinded to the treatments. After echocardiography, the animals were sacrificed. Hearts were weighed and the ventricles were immersed in liquid nitrogen and stored at −70°C for later analysis. Because direct intramyocardial gene delivery targets high expression to the LV but not to other regions of the heart [11], [12], only the LV free wall (i.e. areas of surrounding the injection) were selected for further analyses, except that interventricular septum was used for specific analyses.

### Extraction of Cytoplasmic Protein and Western Blotting

To extract the cytoplasmic protein, the LV free wall tissue (the area surrounding the injection site) was broken and reduced to a powder in liquid nitrogen [11]. The thawed powder was homogenized in a lysis buffer (20 mmol/l Tris (pH 7.5), 10 mmol/l NaCl, 0.1 mmol/l EDTA, 0.1 mmol/l EGTA, 1 mmol/l β-glycerophosphate, 1 mmol/l Na_3_VO_4_, 2 mmol/l benzamidine, 1 mmol/l PMSF, 50 mmol/l NaF, 1 mmol/l DTT and 10 µg/ml each of leupeptin, pepstatin and aprotinin). The resulting tissue homogenates were centrifuged at 2000 rpm for 1 minute at +4°C. The cytosolic fraction was separated out by centrifugation at 12500 rpm for 20 minutes. Protein concentrations were determined by Bio-Rad Laboratories Protein Assay.

For western blot analysis, 30 µg protein was subjected to SDS-PAGE and separated proteins were electrically transferred to nitrocellulose membranes. After blocking the nonspecific background in 5% non-fat milk, nitrocellulose membranes were incubated at +4°C overnight with anti-(P)RR, antiphospho-heat shock protein 27 (HSP27), anti-phospho-p38 mitogen-activated protein kinase (MAPK), anti-phospho-ERK1/2, anti-HSP27, anti-p38 MAPK, anti-ERK1/2, anti-Wnt-3, anti-β-catenin, anti-V-ATPase A1, anti-frizzled-8, anti- connective tissue growth factor (CTGF) and GAPDH. After washing the filters were incubated for 1 hour with an HRP-conjugated anti-rabbit, anti-mouse or anti-goat secondary antibody. Antibodies were obtained from Novus Biologicals (Littleton, CO, USA), Santa Cruz Biotechnology (Santa Cruz, CA, USA), Cell Signaling Technology (Beverly, MA, USA), BD Transduction Laboratories (Lexington, KY, USA) and Millipore (Temecula, CA, USA).

For a second western blot, the membranes were stripped for 30 min at +60°C in stripping buffer containing 62.5 mmol/l tris (pH 6.8), 2% SDS, and 100 mmol/l mercapthoethanol. The protein amounts were detected by enhanced chemiluminescence. The films were scanned and analyzed with Quantity One software (Bio-Rad Laboratories, Hercules, CA, USA).

### Isolation and Analysis of RNA

The RNA was extracted from the left ventricular free wall tissue, from the area surrounding the injection site by using the guanidine-thiocyanate-CsCl method [10]. ANP, cardiac α-actin, CTGF, type I collagen-α (ColIα1), fibroblast growth factor-2 (FGF-2), fibronectin-1, LacZ, α-myosin heavy chain (α-MHC), β-MHC, matrix metalloproteinase-2 (MMP-2), MMP-9, plasminogen activator inhibitor-1 (PAI-1), (P)RR, sarcoplasmic reticulum Ca^2+^ ATPase 2 (SERCA2), skeletal α-actin, TGFβ1, vascular endothelial growth factor (VEGF) and 18S mRNA levels were analyzed by the RT-PCR using TaqMan chemistry on an ABI 7300 Sequence Detection System (Applied Biosystems) as previously described [21]–[24]. The sequences of the forward and reverse primers and for fluorogenic probes for RNA detection are shown in the Table 1. The results were normalized to 18S RNA quantified from the same samples.

**Table 1:** Forward and reverse primer and fluorogenic probe sequences used for real time quantitative RT-PCR analysis.

Gene		Sequence
ANP	Forward	GAAAAGCAAACTGAGGGCTCTG
	Reverse	CCTACCCCCGAAGCAGCT
	Fluorogenic probe	TCGCTGGCCCTCGGAGCCT
Cardiac α-actin	Forward	GGGCCCTCCATTGTCCA
	Reverse	GCACAATACTGTCGTCCTGAGTG
	Fluorogenic probe	CGCAAGTGCTTCTGAGGCGGCTAC
CTGF	Forward	CGCCAACCGCAAGATTG
	Reverse	CACGGACCCACCGAAGAC
	Fluorogenic probe	CACTGCCAAAGATGGTGCACCCTG
ColIα1	Forward	CCCCTTGGTCTTGGAGGAA
	Reverse	GCACGGAAACTCCAGCTGAT
	Fluorogenic probe	CTTTGCTTCCCAGATGTCCTATGGCTATGATG
FGF-2	Forward	CCCGGCCACTTCAAGGAT
	Reverse	GATGCGCAGGAAGAAGCC
	Fluorogenic probe	CCAAGCGGCTCTACTGCAAGAACGG
Fibronectin-1	Forward	GCGAGGCAGGATCAGCTG
	Reverse	CCAATCTTGTAGGACTGACCCC
	Fluorogenic probe	ACCATTGCAAATCGCTGCCATGAA
LacZ	Forward	TCTGTATGAACGGTCTGGTCTTTG
	Reverse	TGCTTCCGTCAGCGCTG
	Fluorogenic Probe	CGACCGCACGCCGCAT
α-MHC	Forward	GCAGAAAATGCACGATGAGGA
	Reverse	CATTCATATTTATTGTGGGATAGCAAC
	Fluorogenic probe	TAACCTGTCCAGCAGAAAGAGCCTCGC
β-MHC	Forward	GCTACCCAACCCTAAGGATGC
	Reverse	TCTGCCTAAGGTGCTGTTTCAA
	Fluorogenic probe	TGTGAAGCCCTGAGACCTGGAGCC
MMP-2	Forward	CATGAAGCCTTGTTTACCATGG
	Reverse	TGGAAGCGGAACGGAAACT
	Fluorogenic probe	TGGCAATGCTGATGGACAGCCC
MMP-9	Forward	CCGCCAACTATGACCAGGATAA
	Reverse	AGTTGCCCCCAGTTACAGTGA
	Fluorogenic probe	TGTATGGCTTCTGTCCTACTCGAGCCGA
PAI-1	Forward	GCTGACCACAGCAGGGAAA
	Reverse	GTGCCCCTCTCACTGATATTGAA
	Fluorogenic probe	CCCGGCAGCAGATCCAAGATGCTAT
(P)RR	Forward	CTTGCTGTGGGCAACCTATTC
	Reverse	CTACCCCCTTCACTGTCACCAT
	Fluorogenic probe	ACCGGCCCCGGGCTACCAT
Serca2	Forward	CAGCCATGGAGAACGCTCA
	Reverse	CGTTGACGCCGAAGTGG
	Fluorogenic probe	ACAAAGACCGTGGAGGAGGTGCTGG
Skeletal α-actin	Forward	TCCTCCGCCGTTGGCT
	Reverse	AATCTATGTACACGTCAAAAACAGGC
	Fluorogenic probe	CATCGCCGCCACTGCAGCC
TGFβ1	Forward	CATCGACATGGAGCTGGTGA
	Reverse	TTGGACAGGATCTGGCCAC
	Fluorogenic probe	ACGGAAGCGCATCGAAGCCATC
VEGF	Forward	GATCCGCAGACGTGTAAATGTTC
	Reverse	TTAACTCAAGCTGCCTCGCC
	Fluorogenic probe	TGCAAAAACACAGACTCGCGTTGCA
18S	Forward	TGGTTGCAAAGCTGAAACTTAAAG
	Reverse	AGTCAAATTAAGCCGCAGGC
	Fluorogenic probe	CCTGGTGGTGCCCTTCCGTCA

ANP, atrial natriuretic peptide; CTGF, connective tissue growth factor; ColIα1, collagen Iα1; FGF-2, fibroblast growth factor-2; α-MHC, α-myosin heavy chain; β-MHC, β-myosin heavy chain; MMP-2, matrix metalloproteinase-2, MMP-9, matrix metalloproteinase-9; PAI-1, plasminogen activator inhibitor-1; (P)RR, (pro)renin receptor; Serca2, Sarcoplasmic reticulum Ca2+ ATPase, TGFβ1, transforming growth factor-β1; VEGF, Vascular endothelial growth factor; RT-PCR, real time quantitative reverse transcription-PCR.

### Zymography

For gelatin zymography analysis, 17 µg protein was subjected to SDS-Page. Gelatin zymography was performed as described previously [25].

### Histology, Immunohistochemistry and Image Analysis

For histological analysis, hearts were fixed in 10% buffered formalin solution. Transversal sections of hearts were embedded in paraffin, and 5-µm sections were cut. Sections were cut from the mid-section of the heart, at the level of the papillary muscles. Samples from different animals were obtained in an identical way and from the corresponding sites in order to make the samples fully comparable. One block and section per heart taken from the same site in all samples was used for staining. Sections were stained with hematoxylin and eosin or Massons's trichrome to examine the fibrotic area of the left ventricle at 1 week and 2 weeks after intramyocardial injection of adenoviral construct expressing LacZ or (P)RR. Previously we have studied the local response to adenovirus-mediated gene transfer by measuring the fibrotic area in the left ventricle at 2 weeks after intramyocardial injection of adenoviral construct expressing LacZ and PBS-based buffer (3%-iodixanol-PBS) as well as from the hearts with needle-stick (no injection of fluid) and non-injected hearts. The results demonstrated that the degree of fibrosis did not differ between PBS-based buffer- and LacZ-injected hearts, but tended to be higher in these groups than in non-injected hearts [15].

To assess cardiomyocyte hypertrophy, cross sectional area of cardiomyocytes was calculated from five correspondingly located fields per sample (3 from epicardial and 2 from endocardial side of the left ventricle). Cross sectional area of ten cells per field was measured using the Nikon NIS_Elements BR 2.30 software.

To detect apoptotic cells, in situ labeling of the 3′-ends of the DNA fragments generated by apoptosis-associated endonucleases was performed using the ApopTag in situ apoptosis detection kit (Oncor, Gaithersburg, MD, USA), as previously described [11], [26]. Briefly, DNA fragmentation was identified by applying terminal transferase enzyme with digoxigenin-labeled nucleotides. Anti-digoxigenin antibody was used to recognize the digoxigenin-labeled nucleotide chains attached to the 3′-ends of sample DNA. A color reaction was produced with diaminobenzidine and the sections were counterstained with hematoxylin. The apoptotic cells and bodies were counted in 5 high power fields (40× objective) choosing hot spot areas in each sample in order to make the results comparable. To determine whether TUNEL positive cells were cardiomyocytes, immunofluorescence staining was performed with alpha-actinin antibody (ab28052, Abcam Inc., Cambridge, MA, USA) as a cardiomyocyte marker and DAPI (diamidinophenylindole dihydrochloride, Sigma-Aldrich, St. Louis, MO, USA) as a nuclear stain.

To examine the efficiency and localization of the (P)RR gene delivery, the sections were incubated with specific polyclonal anti-(P)RR antibody (NB100-1318, Novus Biologicals, Littleton, CO, USA) at the dilution of 1∶200 3 days after gene transfer. Amino-9-ethylcarbazole (Zymed, South San Francisco, CA, USA) was used a chromogen (producing red color). Specific polyclonal anti-(P)RR antibody was raised against a peptide mapping with an extracellular domain of (P)RR. Immunofluorescence staining was performed to analyze the expression of (P)RR in cardiac cells. The antibodies used were NB100-1318 (Novus Biologicals, Littleton, CO, USA) for (P)RR and anti-prolyl 4-hydroxylase β (MAB2073, Millipore, Temecula, CA, USA) for fibroblasts. Nuclei were stained blue with DAPI (diamidinophenylindole dihydrochloride, Sigma, St Louis, MO, USA). To identify cells undergoing division, immunohistochemical labeling of nuclear Ki-67 antigen was performed by using monoclonal mouse anti-rat Ki-67 antigen antibody (DakoCytomation, Glostrup, Denmark). The whole left ventricle was scanned and stained cells were counted from high power fields (40×) choosing 5 hot spot areas in each sample. Pecam-1 (sc-1506-R, Santa Cruz Biotechnology) was used to stain endothelial cells. The number of capillaries was calculated from five representative high power fields (40×) from the left ventricle of each section; 3 from epicardial and 2 from endocardial side of the left ventricle were selected. The primary antibodies were detected by peroxidase conjugated EnVision Detection Kit system (DakoCytomation, Denmark) and the samples were counterstained with haematoxylin. All measurements were performed blinded by persons, who were not aware of the treatments.

### X-gal staining

Hearts were rinsed in PBS and fixed in PBS containing 4% paraformaldehyde for 1 h at 4°C, washed three times in rinse buffer (100 mM sodium phosphate pH 7.3, 2 mM MgCl_2_, 0.01% sodium deoxycholate, 0.02% NP-40) for 30 min, and incubated with 1 mg/ml X-gal (5-Bromo-4-chloro-3-indolyl-beta-D-galactopyranoside) in reaction buffer (5 mM K_3_Fe(CN)_6_, 5 mM K_4_Fe(CN)_6_xH_2_O, 100 mM sodium phosphate pH 7.3, 2 mM MgCl_2_, 0.01% sodium deoxycholate, 0.02% NP-40) at +37°C for 5 h, and post-fixed overnight in 10% formalin.

### Immunoprecipitation

Samples with 300 µg of total protein were incubated with 10 µl of promyelocytic zinc-finger protein (PLZF) (SantaCruz Biotechnology, Santa Cruz, CA, USA) antibody overnight with continuous rocking at +4°C and subsequently conjugated with protein G-agarose beads (30 µl/sample) (SantaCruz Biotechnology, Santa Cruz, CA, USA) for 3 hours with continuous rocking at +4°C. The absence of primary antibody in a parallel reaction mix served as a negative control. The beads were collected by centrifugation, washed 5 times in the lysis buffer, and finally boiled for 5 min in sodium dodecyl sulphate, resolved by SDS-PAGE and transferred to Optitran BAS 85 nitrocellulose membranes. The membranes were blocked in Odyssey Blocking Buffer – TBS (1∶1) and then incubated with specific polyclonal anti-(P)RR antibody (Novus Biologicals Littleton, CO, USA) at a concentration of 1∶100 in Odyssey Blocking Buffer–TBS solution overnight at +4°C. The following day (P)RR antibody binding was detected with Alexa Fluor goat anti-mouse IgG at a 1∶3000 dilution. The chemiluminescence was detected using Odyssey Infrared Detection. The bands were quantified with Quantity One software.

### Statistics

Results are expressed as mean±SEM. Statistical analyses were performed using PASW Statistics version 17.0 (SPSS Inc., Chicago, IL, USA). Because all variables were normally distributed variables, statistical significance was evaluated by one-way ANOVA followed by a least significant difference (LSD) post hoc test for multiple comparisons. Student's t-test was used for comparison between two groups. A *P*-value of <0.05 was considered statistically significant.

## Results

### Augmentation of LV (P)RR Levels by Adenoviral Gene Delivery

**Figure 1:**
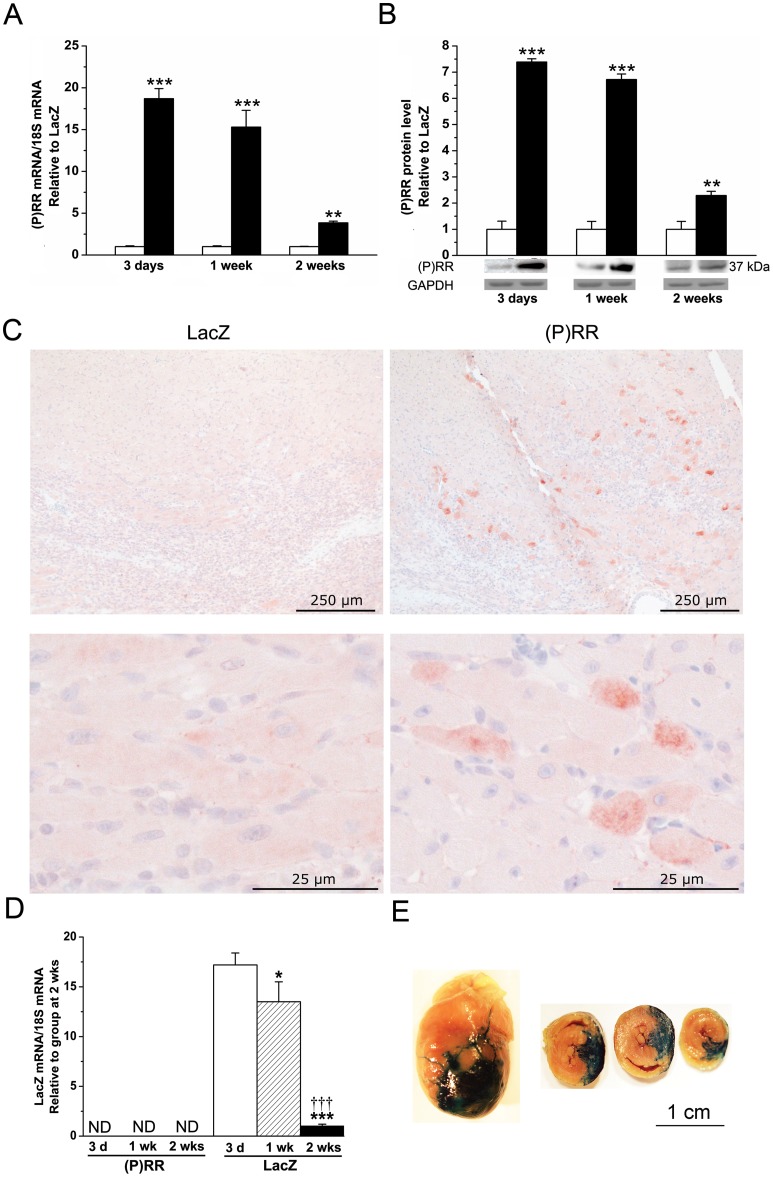
Cardiac-specific activation of (P)RR by adenoviral gene delivery into the left ventricle. **A**, (P)RR mRNA levels measured by RT-PCR, and **B**, (P)RR protein levels assessed by Western Blot analyses from the LV tissue samples 3 days, 1 week and 2 weeks after (P)RR gene delivery. Bands were detected from the same gel. GAPDH was used as a loading control for Western Blot. The results are expressed as mean±SEM (n = 5 to 10). **P<0.01, ***P<0.001 versus LacZ (Student's t-test). Open bars represent LacZ and solid bars (P)RR. **C**, Efficiency of the gene transfer was confirmed by immunohistochemical staining against (P)RR at day 3 after gene transfer. Representative images from LV anterior wall are shown. **D**, LacZ mRNA levels were measured by RT-PCR. The results are expressed as mean±SEM (n = 5 to 10). ND indicates not detectable. *P<0.05, ***P<0.001 versus at 3 days; †††*P*<0.001 versus at 1 week (1-way ANOVA followed by least significance difference post hoc test). **E**, X-gal staining demonstrating localization and efficiency of gene transfer. A large segmental staining area in anterior wall of LV of LacZ-injected hearts was observed at day 3 after gene transfer.

**Figure 2:**
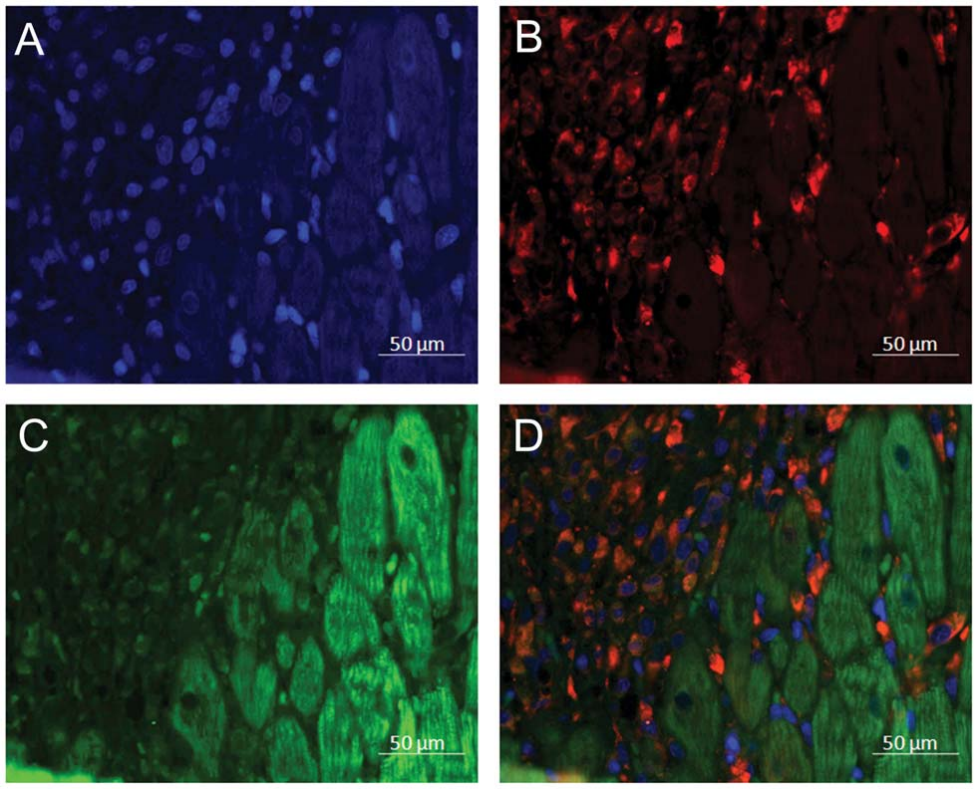
(P)RR is located into the cardiac myocytes in the rat heart. Immunofluorescence staining of LV anterior wall at day 3 after gene transfer showing (P)RR predominantly in cardiomyocytes. **A**, DAPI (blue), **B**, prolyl 4-hydroxylase β staining for fibroblasts (red), **C**, (P)RR (green) and **D**, Merge. Representative image is shown.

To study the direct myocardial effects of (P)RR, we established an *in vivo* gene transfer protocol to locally increase (P)RR levels in the normal adult rat left ventricle. Different doses of adenoviral constructs were first tested to increase LV (P)RR mRNA levels at 2 weeks closely to those observed in experimental models of stroke-prone spontaneously hypertensive rat hearts [16] and with congestive heart failure due to coronary ligation [17]. A clear activation of gene expression, evaluated by quantitative real-time polymerase chain reaction (RT-PCR), as well as an increase in protein concentrations, measured by Western blot, was noted when rat (P)RR expressing adenoviral constructs were injected into the LV free wall at 1×10^9^ infectious units in a 100 µl injection volume (Figure 1A and 1B). (P)RR protein levels at 2 weeks were quantitatively equal to the (P)RR protein levels (about 2- to 3-fold higher compared to controls) observed post-infarction in rat hearts and in patients with dilated cardiomyopathy [18], and in the hearts of diabetic rats [27]. When the efficiency and localization of the (P)RR gene delivery was studied by immunohistochemistry, analysis of (P)RR-Ad5 injected animals showed local and augmented segmental granular staining in the cardiomyocytes of the LV anterior wall compared to LacZ-treated hearts (Figure 1C). LacZ-Ad5 vector is the most frequently employed control vector, because LacZ encodes the protein (β-galactosidase) also used to standardize virus production. This vector does not affect myocardial function as assessed by systolic wall thickening using ultrasonic crystals [28]. LacZ mRNA levels (Figure 1D) were highest at day 3 after LacZ-injection and decreased significantly thereafter during the follow-up period. LacZ was not detectable by RT-PCR in hearts of animals injected with adenovirus expressing (P)RR. X-gal staining demonstrated a large segmental staining area in anterior wall of the LV of LacZ-injected hearts at day 3 after gene transfer (Figure 1E). The time course for LacZ expression following direct intramyocardial injection of LacZ-Ad5 vector similar to ours has been reported previously [28], [29]. Immunofluorescence staining further confirmed that (P)RR was localized predominantly into the cardiac myocytes in the adult rat heart (Figure 2). Very recently, using confocal microscopy, site-specific markers and transmission electron microscopy, (P)RR was reported to be located mainly in T-tubules in rat hearts [27].

### Local Myocardial (P)RR Gene Delivery Deteriorates Cardiac Function

**Figure 3:**
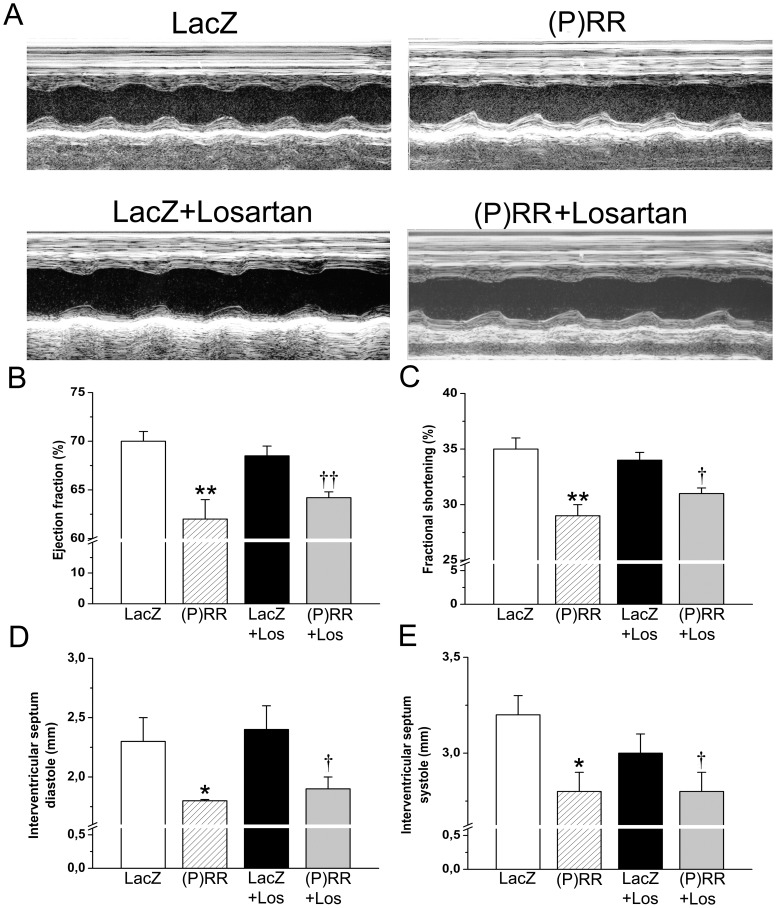
Intramyocardial (P)RR gene delivery deteriorates cardiac function in normal heart. Adenoviral gene construct expressing (P)RR and LacZ were injected into LV free wall and losartan (Los) 400 µg/kg/h was administered via osmotic minipumps. Echocardiographic measurements were performed at 1 week and 2 weeks after gene transfer and losartan treatment. **A**, Representative M-mode images are shown. Treatment of normal rat hearts with (P)RR-overexpressing vector significantly decreased EF (**B**) and FS (**C**) at 2 weeks, and intraventricular septum diastolic (**D**) and systolic thickness (**E**) at 1 week in the presence of losartan. The results are mean±SEM (n = 8 to 10). *P<0.05, **P<0.01 versus LacZ; †*P*<0.05, ††*P*<0.01 versus LacZ with losartan (1-way ANOVA followed by least significance difference post hoc test).

To evaluate the effect of (P)RR gene delivery on cardiac function, we performed echocardiography. Left ventricular EF and FS deteriorated (Figure 3A through C, Table 2), and the intraventricular septum diastolic and systolic thickness were significantly reduced in response to (P)RR gene transfer (Figure 3D and 3E). To test whether the worsening of cardiac function and structure by (P)RR gene overexpression was mediated by Ang II, we infused an AT_1_ receptor blocker losartan. Strikingly, LV EF and FS declined, and the intraventricular septum diastolic and systolic thickness decreased significantly by (P)RR gene transfer also in losartan-treated animals (Figure 3A through 3E). Losartan treatment alone did not affect cardiac function (Figure 3).

**Table 2:** Effect of intramyocardial (P)RR gene delivery on cardiac function in normal adult rat heart.

Variable	Duration	LacZ	(P)RR
**Ejection fraction (%)**	3 days	76±2	76±3
	1 week	70±2	73±2
	2 weeks	70±1	62±1**
**Fractional shortening (%)**	3 days	40±2	40±3
	1 week	35±2	37±2
	2 weeks	35±1	29±1**
**Interventricular septum**			
diastole (mm)	3 days	2.0±0.2	1.9±0.2
	1 week	2.3±0.2	1.8±0.1*
	2 weeks	2.0±0.1	1.8±0.1
systole (mm)	3 days	3.2±0.1	3.1±0.3
	1 week	3.2±0.1	2.8±0.1*
	2 weeks	2.9±0.1	2.4±0.1*
**Left ventricle**			
diastole (mm)	3 days	7.5±0.2	7.9±0.2
	1 week	7.2±0.3	7.3±0.2
	2 weeks	8.2±0.3	8.2±0.2
systole (mm)	3 days	4.5±0.2	4.7±0.3
	1 week	4.7±0.2	4.6±0.2
	2 weeks	5.3±0.2	5.8±0.1

Adenoviral gene construct expressing (P)RR and LacZ were injected into LV free wall and echocardiographic measurements were performed at 3 days, 1 week and 2 weeks after gene transfer. The results are expressed as mean±SEM (n = 5 to 10).

* *P*<0.05,

** P<0.01 versus LacZ (Student's t-test).

### (P)RR Triggers Angiotensin II-Independent Extracellular Matrix Remodeling in Normal Adult Rat Heart

**Figure 4:**
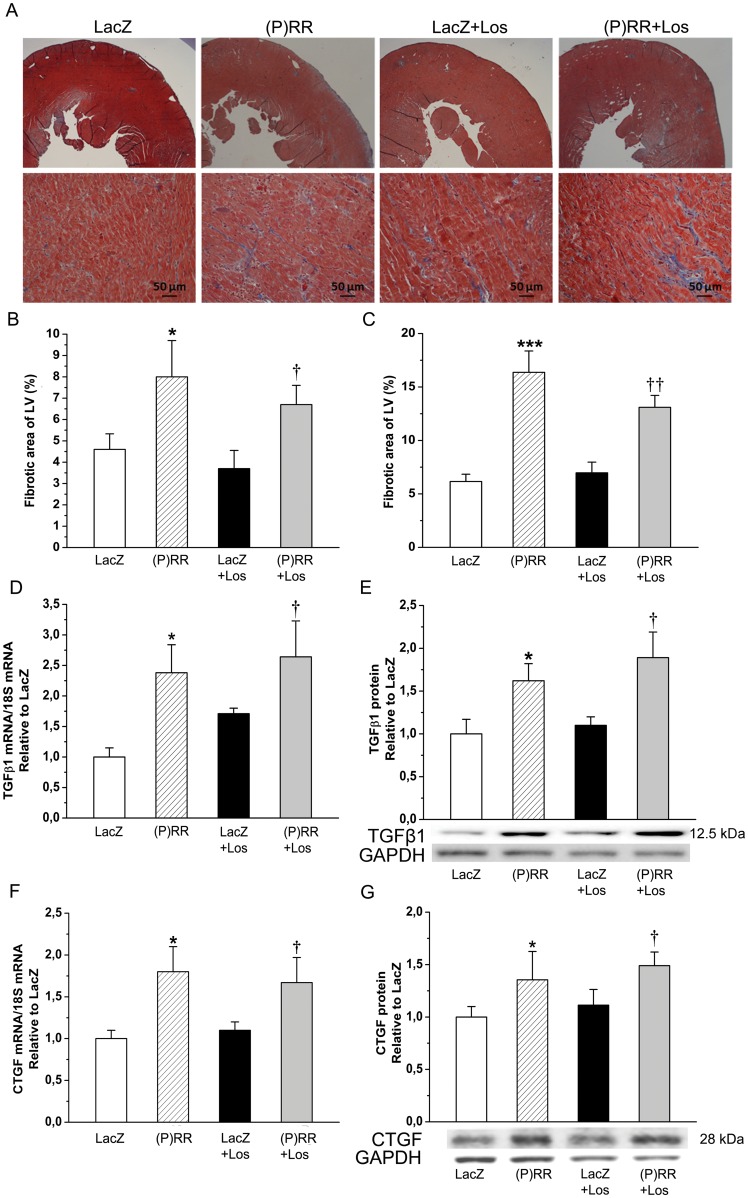
Local (P)RR gene delivery increases myocardial fibrosis and expression of pro-fibrotic genes. Paraffin-embedded histological sections were stained with Masson's trichrome to define the area of fibrosis by computerized methods at 1 week and 2 weeks after (P)RR gene delivery and losartan (Los) treatment. **A**, Representative images are shown at 2 weeks. **B**, (P)RR-treated hearts show significantly more LV fibrosis than LacZ-treated hearts at 1 week and **C**, at 2 weeks. **D**, Transforming growth factor-β1 (TGFβ1) mRNA and **E**, TGFβ1 protein levels at 1 week, and **F**, Connective tissue growth factor (CTGF) mRNA at 2 weeks. **G**, CTGF protein levels at 2 weeks. The results are expressed as mean±SEM (n = 5 to 10). *P<0.05, ***P<0.001 versus LacZ; †P<0.05, ††*P*<0.01 versus LacZ with losartan (1-way ANOVA followed by least significance difference post hoc test).

**Figure 5:**
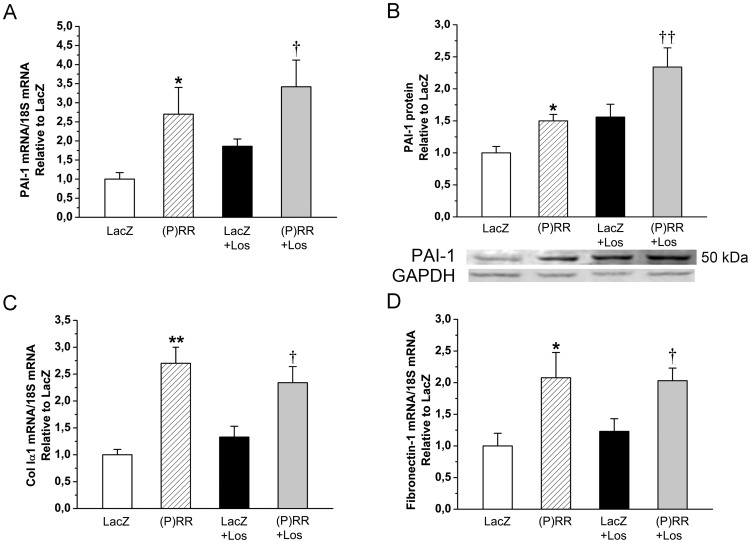
Intramyocardial (P)RR gene delivery increases plasminogen activator inhibitor-1 (PAI-1), collagen 1α1 (Col Iα1), fibronectin-1 gene expressions independent of Ang II formation. **A,** PAI-1 mRNA, **B,** PAI-1 protein, **C,** Col Iα1, **D,** Fibronectin-1. The results are expressed as mean±SEM (n = 8 to 10). *P<0.05, **P<0.01 versus LacZ; *†P*<0.05, *††P*<0.01 versus LacZ with losartan (Los) (1-way ANOVA followed by least significance difference post hoc test).

**Figure 6:**
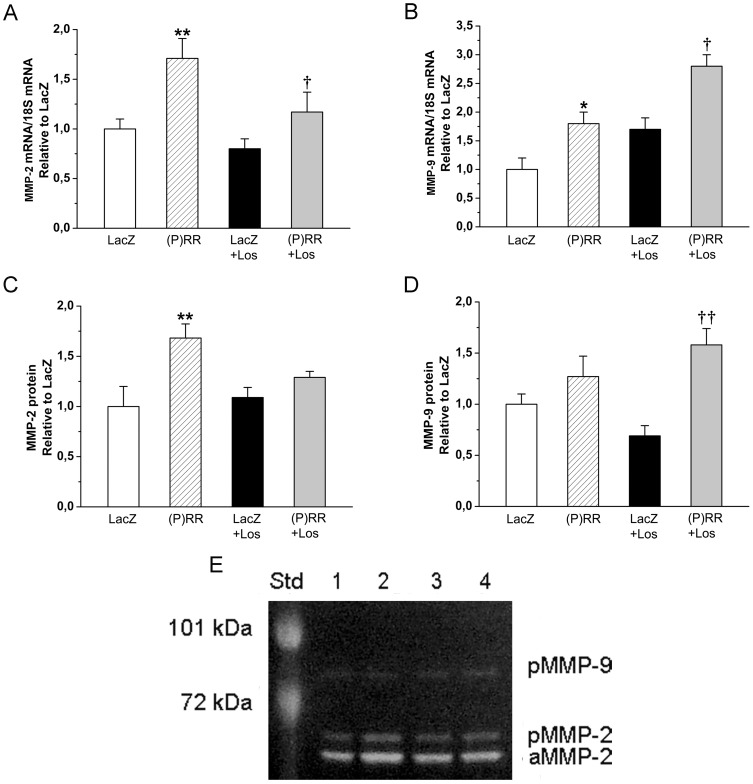
Effects of intramyocardial (P)RR gene delivery on matrix metalloproteinase-2 (MMP-2) and matrix metalloproteinase-9 (MMP-9) expressions. **A,** MMP-2 mRNA levels at 2 weeks and **B,** MMP-9 mRNA levels at 1 week. Gelatin zymography, **C** for MMP-2 and, **D** for MMP-9. **E,** Representative image for active-MMP-2 (aMMP-2), pro-MMP-2 (pMMP-2) and pro-MMP-9 (pMMP-9) is shown at 2 weeks. Active-MMP-9 was not detected. Protein molecular weight standards (72 kDa and 101 kDa) are shown on the left. The results are expressed as mean±SEM (n = 8 to 10). *P<0.05, **P<0.01 versus LacZ; *†P*<0.05, *††P*<0.01 versus LacZ with losartan (Los) (1-way ANOVA followed by least significance difference post hoc test).

**Figure 7:**
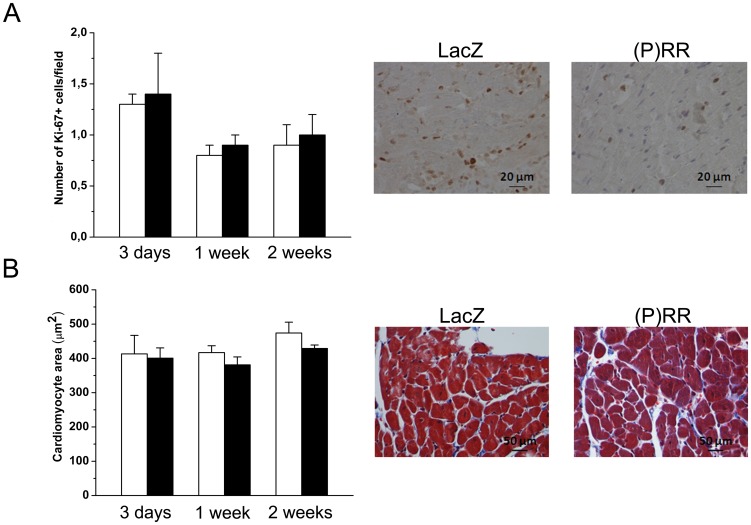
Myocardial cell proliferation and cardiomyocyte mean cross sectional area in the left ventricle at 2 weeks after local (P)RR gene delivery. **A,** Immunohistochemical staining against Ki-67 was performed to determine the effect of (P)RR gene transfer on cellular proliferation. The whole left ventricle was scanned and stained cells were counted from high power fields (40×) choosing 5 hot spot areas in each sample. **B,** Paraffin-embedded histological sections were stained with Masson trichrome to define the cardiomyocyte cross sectional area from five correspondingly located fields per sample. Representative images are shown. Open bars represent LacZ and solid bars (P)RR. The results are expressed as mean±SEM (n = 5 to 10). *P* = ns versus LacZ (Student's t-test).

As assessed by staining histological sections with Masson's trichrome (Figure 4A), myocardial fibrosis increased significantly by (P)RR gene delivery at 1 week (Figure 4B) and 2 weeks (Figure 4C). Consistent with this, (P)RR overexpression significantly increased local left ventricular expression of TGFβ1 and CTGF (Figure 4D through 4G). (P)RR gene transfer also augmented the expression of other genes related to extracellular matrix remodeling such as PAI-1, Col Iα1, fibronectin-1 (Figure 5), MMP-2 and MMP-9 (Figure 6). Gelatin zymography analysis showed that (P)RR gene delivery resulted in a statistically significant increase in MMP-2 (pro and active forms; Figure 6C and 6E) and a non-significant increase in MMP-9 (pro form; Figure 6D and 6E) protein levels. Losartan treatment did not prevent fibrosis (Figure 4A through 4C) or the activation of pro-fibrotic and fibrosis-related genes in (P)RR overexpressing hearts (Figure 4D through 4G, Figure 5, Figure 6), except that only a non-significant increase in MMP-2 protein levels in losartan-treated (P)RR overexpressing hearts was noted (Figure 6C and 6E). When cell proliferation and size of cardiomyocytes were determined, no difference in the number of Ki-67+ cells and cardiomyocyte cross sectional area, respectively, between groups was observed (Figure 7).

### Ang II-Dependency of Activation of Cardiac Gene Expression by (P)RR Gene Delivery

**Figure 8:**
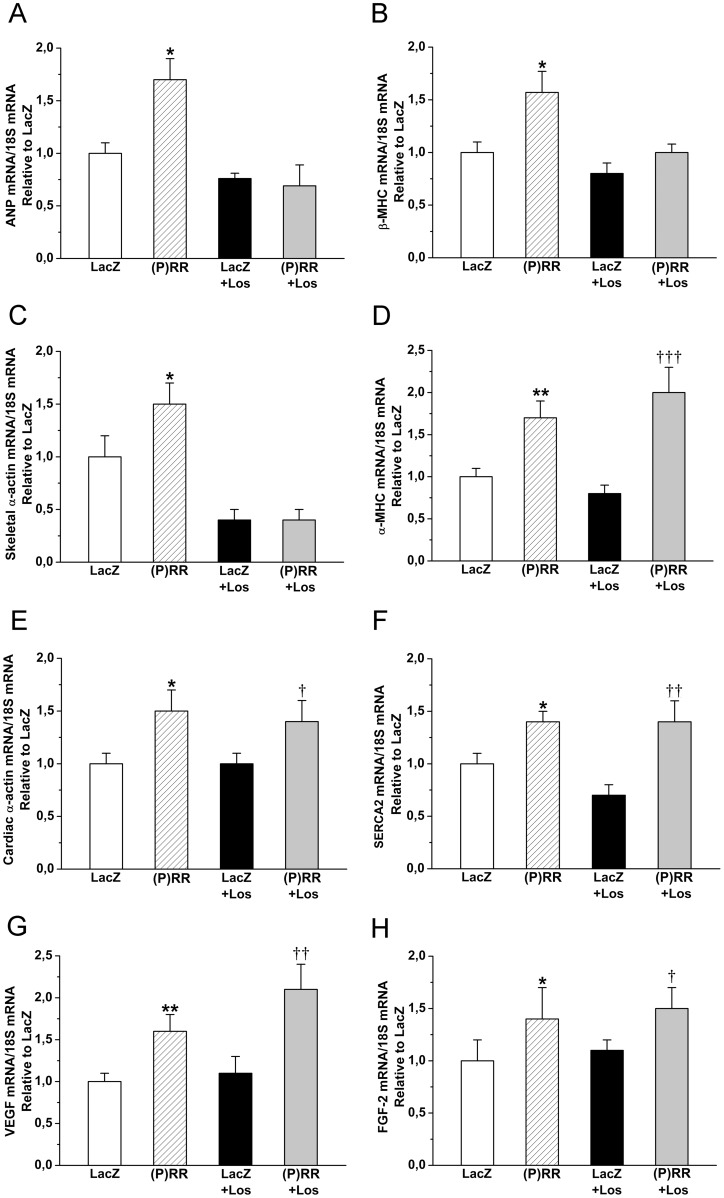
Effect of intramyocardial (P)RR gene delivery on cardiac gene expression without or with the losartan (Los) treatment. **A,** atrial natriuretic peptide (ANP) mRNA, **B,** β-myosin heavy chain (β-MHC) mRNA, **C,** skeletal α-actin mRNA, **D,** α-myosin heavy chain (α-MHC), **E,** cardiac α-actin mRNA, **F,** sarcoplasmic reticulum Ca^2+^ ATPase (SERCA2) mRNA, **G,** vascular endothelial growth factor (VEGF) mRNA at 2 weeks and **H,** fibroblast growth factor-2 (FGF-2) mRNA levels at 1 week. The results are expressed as mean±SEM (n = 8 to 10). *P<0.05, **P<0.01 versus LacZ; *†P*<0.05, *††P*<0.01, *†††P<0.001* versus LacZ with losartan (1-way ANOVA followed by least significance difference post hoc test).

**Figure 9:**
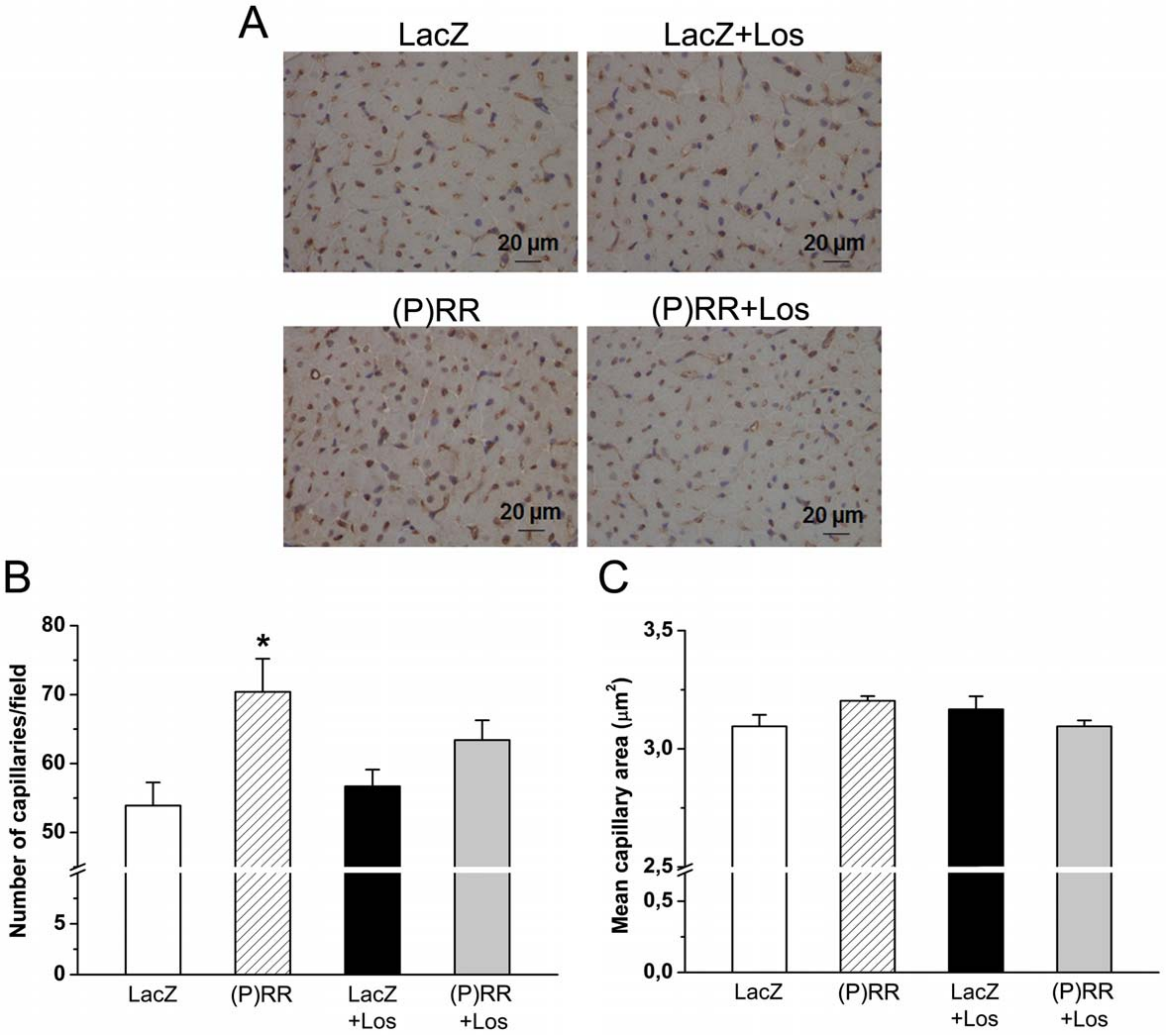
Local (P)RR gene transfer increases mean capillary density in the left ventricle at 1 week. **A,** Representative images from Pecam-1 stained left ventricular sections. **B,** Number of capillaries per field and **C,** mean capillary area with and without losartan (Los) treatment were counted in 5 representative fields in the left ventricle. The results are expressed as mean±SEM (n = 5 to 8). *P<0.05 versus LacZ (1-way ANOVA followed by least significance difference post hoc test).

Next, we examined whether the Ang II-dependent or Ang II-independent signaling pathways result in the activation of specific genes related to myocardial remodeling. Overexpression of (P)RR produced a significant increase in the LV gene expression of well-established markers of cardiac hypertrophic response such as ANP, β-MHC and skeletal α-actin [30], [31], and losartan treatment significantly reduced the activation of all these genes (Figure 8A through 8C). Also expression of genes involved in the regulation of cardiac contractility, e.g, α-MHC, cardiac α-actin and SERCA2 [31], [32] were significantly augmented by (P)RR gene delivery; however, losartan treatment did not prevent their activation in (P)RR overexpressing left ventricles (Figure 8D through 8F). Furthermore, local (P)RR gene transfer resulted in a statistically significant increase in mRNA levels of angiogenic factors VEGF [33] and FGF-2 [34] these increases being not prevented by losartan administration (Figure 8G and 8H). To investigate the effect of (P)RR gene delivery on coronary angiogenesis, histological sections were immunohistochemically stained against Pecam-1. Local (P)RR gene delivery resulted in a statistically significant increase in capillary density, and there was also a non-significant increase in capillary density in losartan-treated (P)RR overexpressing hearts (Figure 9A and 9B), whereas (P)RR gene transfer had no effect on mean capillary area (Figure 9C).

### (P)RR Activates ERK1/2 and p38 MAPK/HSP27 Pathways

**Figure 10:**
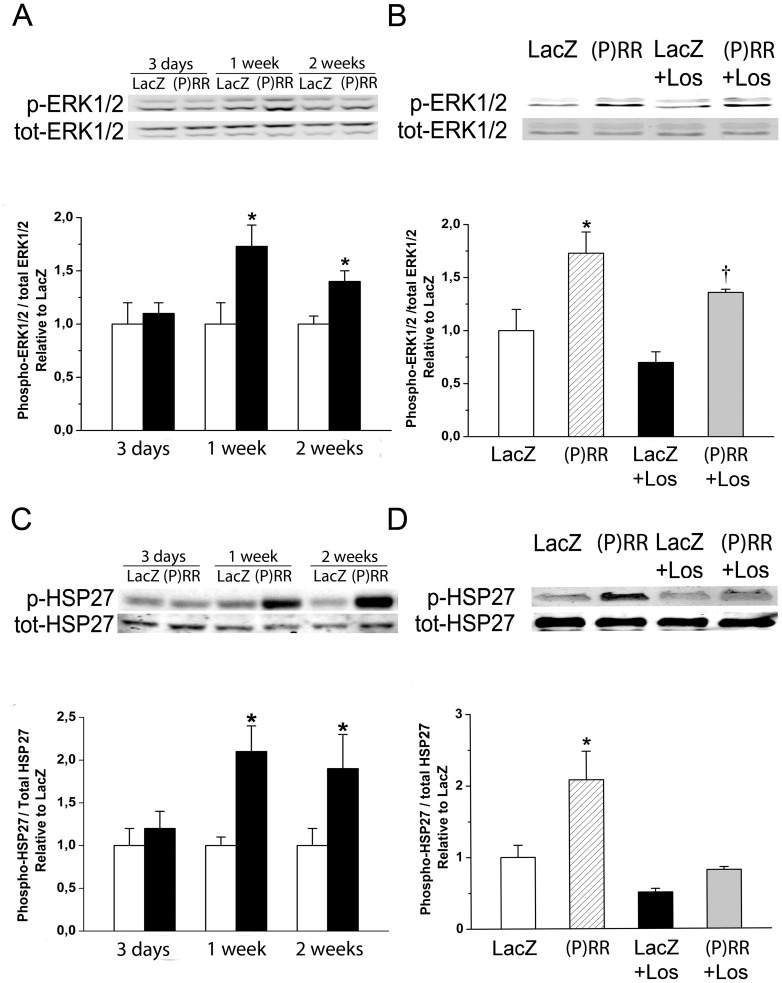
Up-regulation of ERK1/2 and HSP27 phosphorylation. ERK1/2 (**A**) and HSP27 (**C**) phosphorylations 3 days, 1 week and 2 weeks after (P)RR gene delivery. ERK1/2 (**B**) and HSP27 (**D**) phosphorylations at 1 week after (P)RR gene transfer and losartan (Los) treatment. Bands were detected from the same gel. Representative Western blots are shown. The results are mean±SEM (n = 8 to 10). *P<0.05 versus LacZ; *†P<0.05* versus LacZ with losartan (1-way ANOVA followed by least significance difference post hoc test).

**Figure 11:**
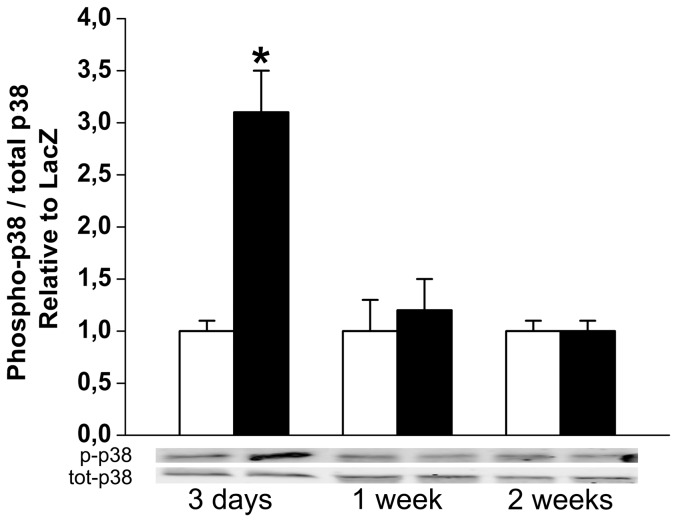
Up-regulation of p38 MAPK phoshorylation by intramyocardial (P)RR gene transfer. p38 MAPK phosphorylation at day 3, 1 week and 2 weeks after (P)RR gene transfer. Bands were detected from the same gel. Representative Western blots are shown. Open bars represent LacZ and solid bars (P)RR. The results are mean±SEM (n = 5 to 10). *P<0.05 versus LacZ (Student's t-test).

**Figure 12:**
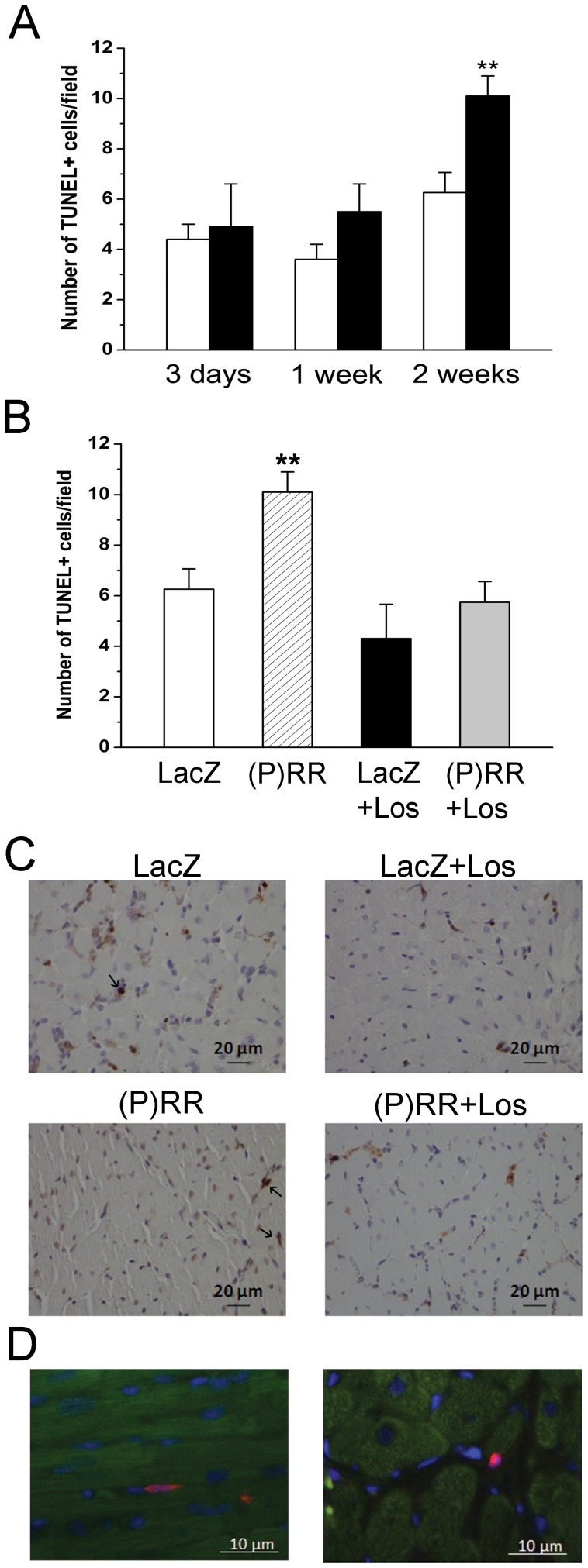
Myocardial apoptotic cell death in the left ventricle after local (P)RR gene delivery. The rate of apoptosis was assessed by TUNEL. The apoptotic cells and bodies were counted in 5 high power fields (40× objective) from the LV free wall regions choosing hot spot areas in each sample in order to make the results comparable. **A,** TUNEL+ cells 3 days, 1 week and 2 weeks after (P)RR gene delivery. Arrows indicate TUNEL+ cells. Open bars represent LacZ and solid bars (P)RR. The results are expressed as mean±SEM (n = 5 to 10). **P<0.01 versus LacZ (Student's t-test). **B,** TUNEL+ cells 2 weeks after (P)RR gene transfer and losartan (Los) treatment. The results are mean±SEM (n = 8 to 10). **P<0.01 versus LacZ (1-way ANOVA followed by least significance difference post hoc test). **C,** Representative images of TUNEL+ cells are shown. **D,** Immunofluorescence staining showing that apoptotic cells were non-cardiomyocytes (Red, TUNEL; green, α-actinin; blue, DAPI).

Originally, Nguyen et al [3] found that in mesangial and vascular smooth muscle cells binding of prorenin to (P)RR induced the phosphorylation of the ERK1/2. Therefore, we assessed the changes of ERK1/2 phosphorylation by Western Blot analyses following (P)RR gene transfer. As shown in Figure 10A, (P)RR gene delivery significantly increased ERK1/2 phosphorylation. Interestingly, infusion of losartan had no effect on the (P)RR gene delivery induced increase in ERK1/2 phosphorylation (Figure 10B). We also observed that (P)RR gene transfer increased heat shock protein 27 (HSP27) (Figure 10C) and p38 MAPK (Figure 11) phosphorylation, the former being significantly attenuated by losartan (Figure 10D). (P)RR gene transfer increased apoptotic cell death at 2 weeks, which was significantly reduced by losartan in (P)RR-overexpressing hearts (Figure 12A through 12C). Double immunofluorescence staining of TUNEL+ cells showed that they were not positive for cardiomyocyte marker alpha-actinin (Figure 12D).

### Effects of (P)RR Gene Transfer on Intraventricular in Normal Adult Rat Heart

**Figure 13:**
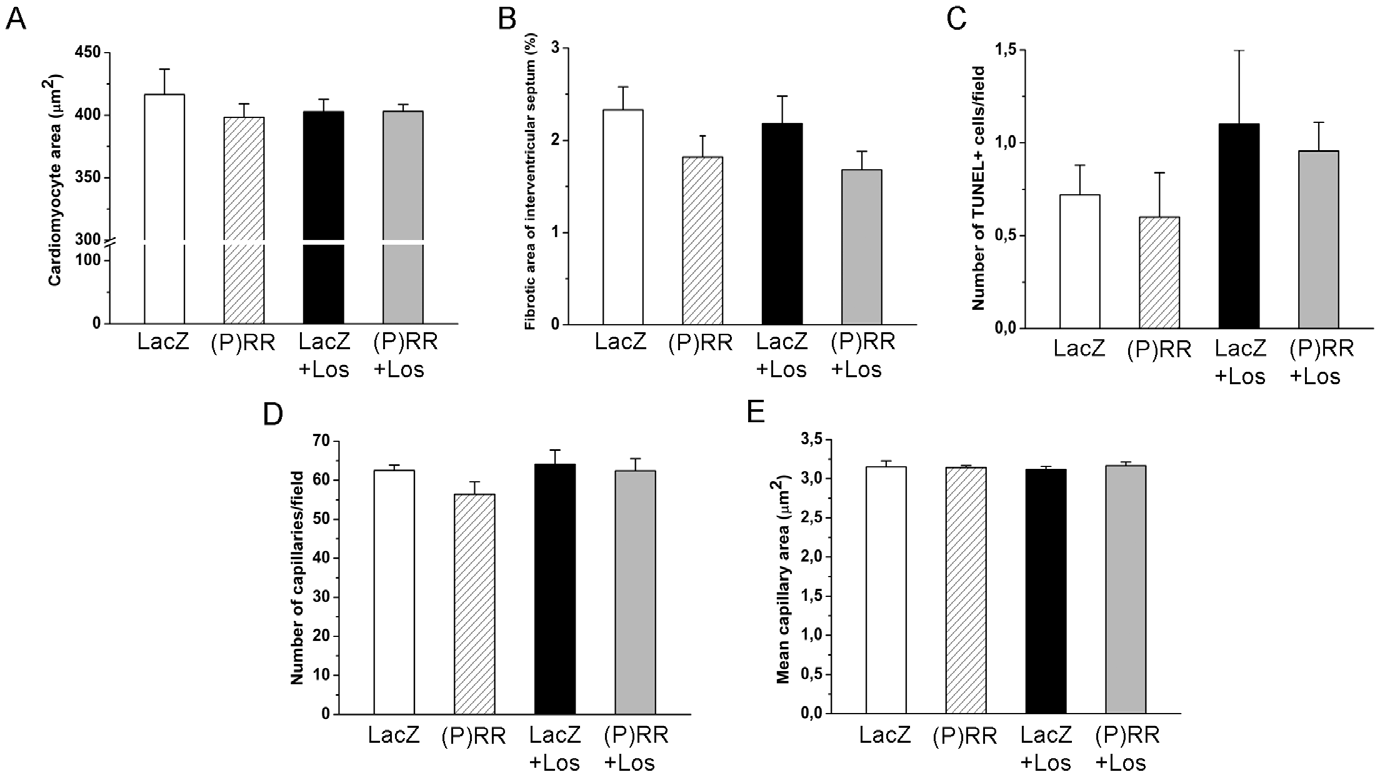
Effects on adenovirus-mediated (P)RR gene transfer on interventricular septum in normal adult rat heart. **A,** Cardiomyocyte cross sectional area, **B,** fibrotic area, **C,** apoptotic cells, **D,** number of capillaries per fields and **E,** mean capillary area with and without losartan (Los) treatment at one week. The results are expressed as mean±SEM (n = 5 to 8). *P = ns* (1-way ANOVA followed by least significance difference post hoc test).

Because echocardiographic measurements provided evidence for the thinning of the interventricular septum by (P)RR gene delivery (Table 2), we performed quantification of fibrosis, apoptotic cells, cardiomyocyte area and coronary angiogenesis in this region. As shown in the Figure 13, the degree of fibrosis, number of TUNEL+ cells, cardiomyocyte cross sectional area or coronary angiogenesis did not statistically significantly differ between LacZ and (P)RR treated groups.

### (P)RR Interaction with PLZF

**Figure 14:**
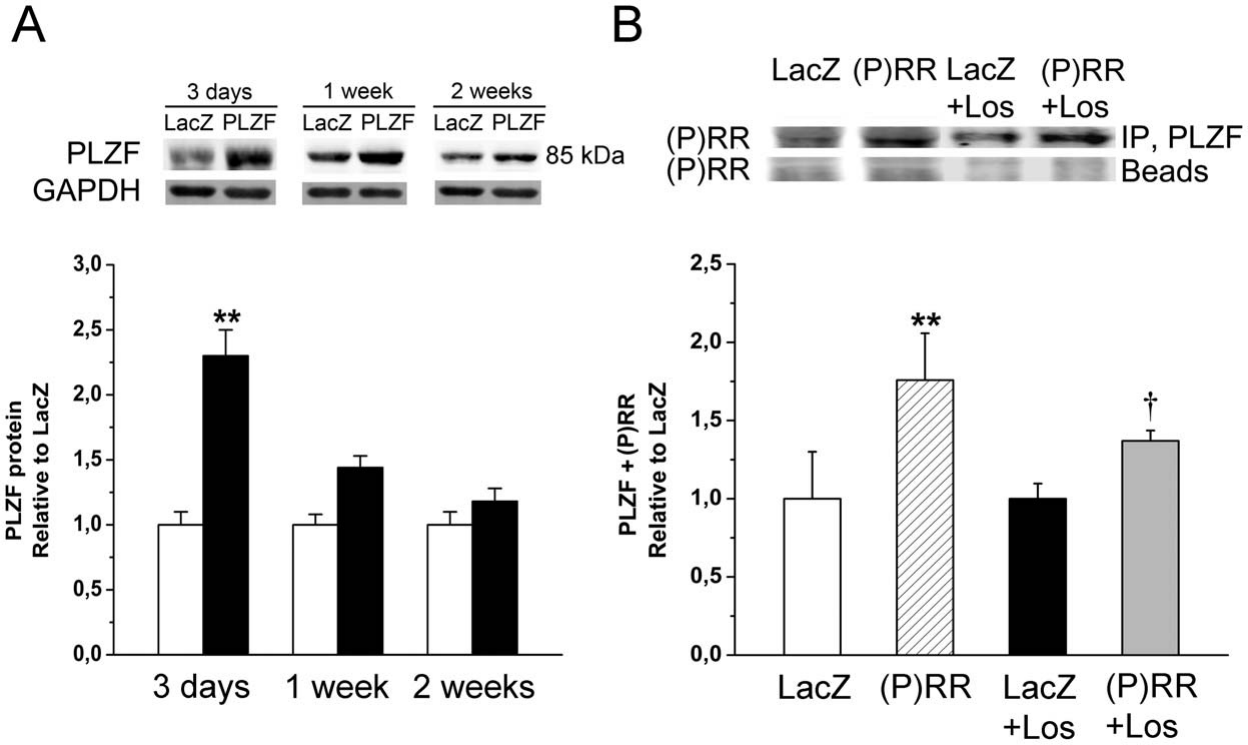
Upregulation of promyelocytic zinc finger protein (PLZF) – prorenin receptor ((P)RR) interaction. **A,** PLZF levels measured by Western Blot analyses from the left ventricle tissue samples after (P)RR gene transfer. Bands were detected from the same gel. GAPDH was used as a loading control for Western Blot. Open bars represent LacZ group and solid bars (P)RR group. The results are expressed as mean±SEM (n = 5 to 10). **P<0.01 versus LacZ (Student's t-test). **B,** (P)RR interaction with PLZF was detected by immunoprecipitation from total protein cell lysates at 2 weeks. Bands were detected from the same gel. Representative immunoblots are shown. The results are mean±SEM (n = 8 to 10). **P<0.01 versus LacZ; *†P<0.05* versus LacZ with losartan (Los) (1-way ANOVA followed by least significance difference post hoc test).

We also examined direct protein-protein interaction between the (P)RR and the transcription factor PLZF after (P)RR gene transfer by immunoprecipitation, because PLZF acts as direct protein interaction partner of the (P)RR [35]. (P)RR overexpression significantly increased direct protein-protein interaction between (P)RR and PLZF at 2 weeks (Figure 14B), whereas protein levels of PLZF increased at day 3 (Figure 14A). Infusion of losartan did not significantly decrease the interaction between (P)RR and PLZF (Figure 14B).

### (P)RR and Wnt-signaling Proteins

**Figure 15:**
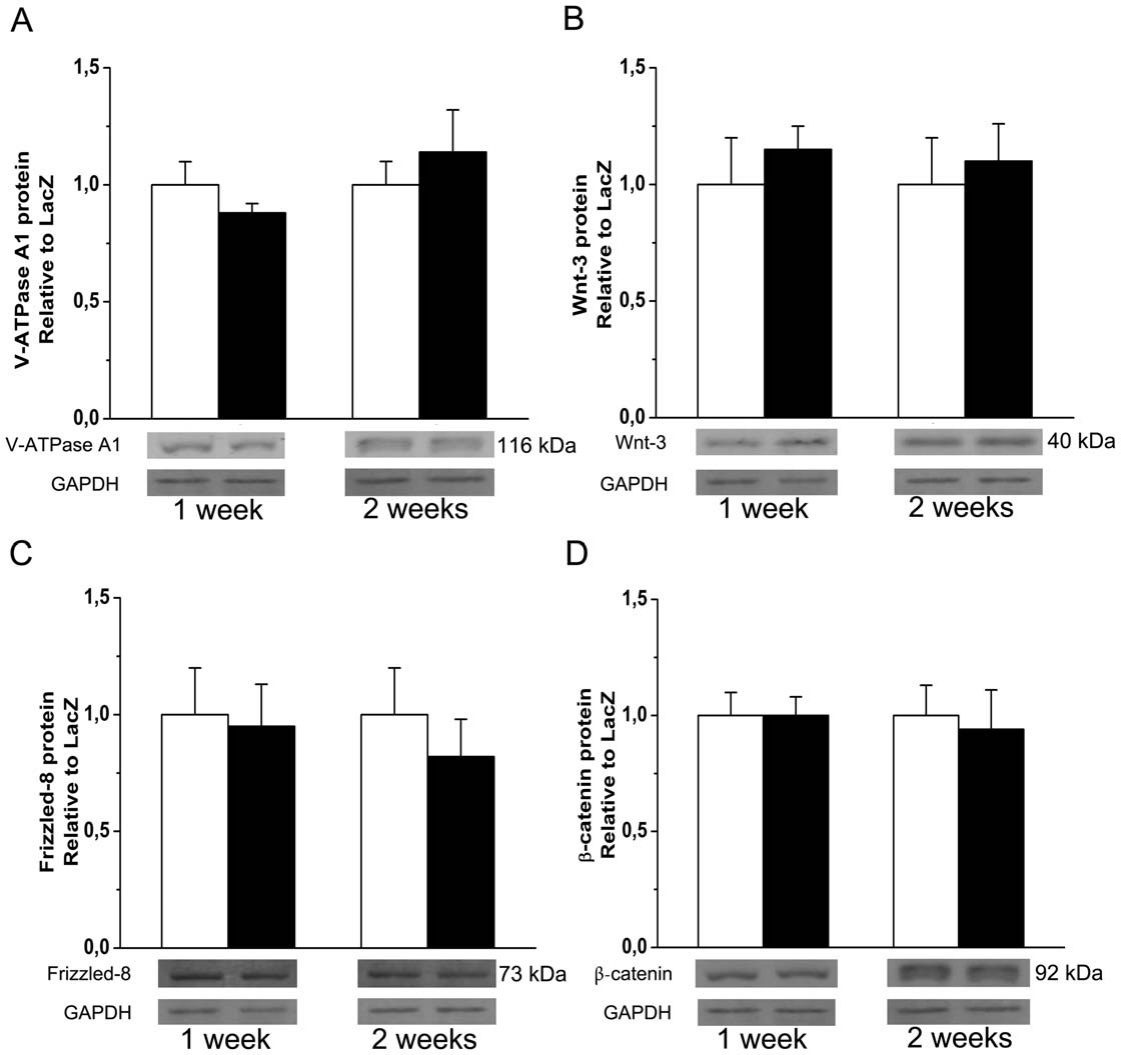
Effect of intramyocardial (P)RR gene delivery on expression of V-ATPase and Wnt-signaling proteins. **A,** V-ATPase A1, **B,** Wnt-3, **C,** Frizzled-8 and **D,** β-catenin protein levels 1 week and 2 weeks after (P)RR gene delivery. Bands were detected from the same gel. GAPDH was used as a loading control for Western Blot. Representative Western blots are shown. Open bars represent LacZ and solid bars (P)RR. The results are mean±SEM (n = 5 to 10). *P* = ns versus LacZ (Student's t-test).

Finally, because (P)RR has been linked to Wnt signaling and V-ATPase [8], [9], we investigated the effects of myocardial (P)RR gene delivery on protein levels of Wnt-3, β-catenin, Frizzled-8 and V-ATPase A1. As shown in Figure 15, the levels of all these proteins remained unchanged in (P)RR-treated hearts suggesting that (P)RR gene transfer did not influence Wnt signaling and V-ATPase pathways.

## Discussion

Recently (P)RR has been characterized a key component of the local RAS [2]. By binding to (P)RR, prorenin becomes catalytically active, leading to generation of Ang II. Renin and prorenin binding to (P)RR also results in the activation of a cascade of intracellular signalling pathways followed by up-regulation of expression of pro-fibrotic genes [36]. Ubiquitous overexpression of (P)RR or prorenin show either no cardiac phenotype or hypertensive LV hypertrophy without fibrosis [4]–[7], [36]. Although beneficial effects of handle region peptide (HRP), a putative (P)RR blocker, have been obtained in animal models, clear evidence that this peptide blocks prorenin-(P)RR interaction is lacking [36], [37]. Moreover, the phenotype of ablation of (P)RR, the gene product of *Atp6ap2*, may be attributable to renin- and prorenin-independent vacuolar H^+^-ATPase and Wnt receptor signalling [10]. Therefore, at present the local actions of (P)RR in the adult hearts are uncertain. By using local direct intramyocardial adenovirus mediated gene delivery, we provide here *in vivo* evidence that (P)RR triggers deterioration of cardiac function. Strikingly, infusion of an AT_1_ receptor blocker did not prevent the decrease in ejection fraction or fractional shortening indicating that (P)RR induced worsening of LV function independent of Ang II generation.

Alterations in the extracellular matrix - leading to fibrosis, dilatation and shape change - are well-established components of complex cardiac remodeling process. The overloaded or failing cardiomyocytes signals to fibroblasts and other cells within the matrix through release of factors such as TGFβ1 and CTGF [38]. In the present study, (P)RR gene delivery in the normal adult heart induced deleterious myocardial fibrosis associated with the increased gene expression of TGFβ1 and CTGF as well as collagen Iα1, PAI-1 and fibronectin-1 indicating that (P)RR plays a critical role in hearts undergoing fibrotic remodeling process. Extracellular matrix remodeling includes also activation of collagenolytic enzymes (matrix metalloproteinases) that lead to ventricular dilatation [38], and interestingly, both MMP-2 and MMP-9 gene expressions were increased in (P)RR overexpressing hearts associated with reduced intraventricular septum diastolic and systolic thickness. Like the worsening of cardiac function, fibrosis and the activation of fibrotic and matrix metalloproteinase genes were not prevented by infusion of losartan indicating that (P)RR triggers myocardial extracellular matrix remodeling independent of Ang II generation as well.

In addition to pathological fibrosis, LV remodeling is characterized by increased cardiomyocyte hypertrophy and impaired vascularization. Many pathways can regulate cardiomyocyte hypertrophy, acting through a complex network of intracellular signaling cascades [39], [40], while insufficient growth in capillary density (due to attenuated release of angiogenic factors like VEGFs) relative to increasing muscle mass promotes pathological remodeling [41]. Here we found that (P)RR gene delivery induces distinct activation of the downstream genes involved in cardiomyocyte hypertrophy and angiogenesis: (P)RR induced Ang II-dependent activation of pathological hypertrophy associated genes (ANP, β-MHC and skeletal α-actin) [30], [31], whereas the (P)RR induced augmentation of expression of angiogenic factors VEGF and FGF-2 [33], [34] was Ang II-independent. In agreement with latter finding, myocardial capillary density was increased by (P)RR gene delivery into LV, and non-significantly also in the presence of losartan treatment. Furthermore, consistently with the finding that (P)RR induced worsening of LV function independent of Ang II generation, also the induction of contractility genes (α-MHC, cardiac α-actin and SERCA2) [31], [32] were Ang II-independent. SERCA2 is responsible for calcium reuptake from the cytosol into the lumen of the sarcoplasmic reticulum, and reduced SERCA2 expression, observed consistently in HF, impairs the calcium-handling and contractile functions of the heart [32]. One possibility might be the short-term effect of Ad5-mediated gene transfer in view that e.g. the activation of hypertrophy marker genes such as ANP (observed in the present study) often precedes the development of cardiomyocyte hypertrophy [20], [30], [31].

**Figure 16:**
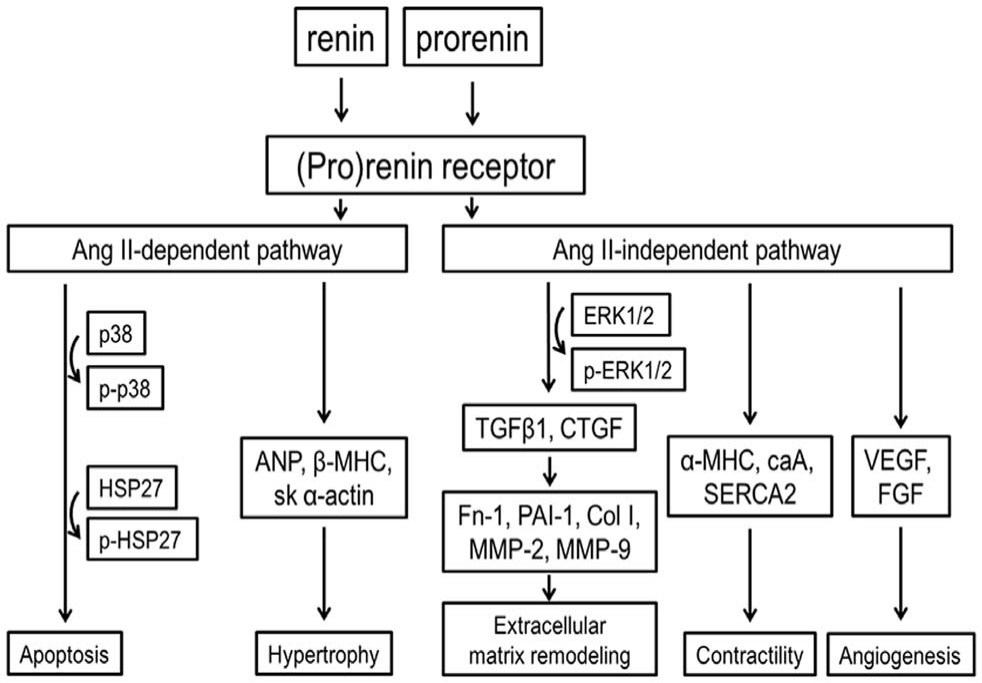
A schematic presentation of Ang II-dependent and Ang II-independent (P)RR pathways. Our results indicate that (P)RR gene delivery induced the phosphorylation of ERK1/2 and p38 MAPK/HSP27 pathways, and had deleterious effects on cardiac function and structure. Furthermore, local (P)RR plays an important role in the regulation of myocardial fibrosis, contractility and angiogenesis via Ang II-independent pathway, and apoptosis and cardiac hypertrophic response via Ang II-dependent pathway.

In most cell types (P)RR activates the phosphorylation of ERK1/2 as well as p38 MAPK/HSP27 pathways [36]. In the present study infusion of losartan had no effect on the (P)RR gene delivery induced increase in ERK1/2 phosphorylation indicating that the activation of ERK1/2 pathway by (P)RR is Ang II-independent. In contrast, losartan significantly attenuated phosphorylation of HSP27 showing that HSP27 activation by (P)RR is at least partly dependent on Ang II. HSP27, through its regulation of actin filament dynamics is suggested to be involved in maintaining the integrity of cell architecture, growth, motility, survival and death [42]. Loss of myocytes in the chronically overloaded heart is believed to contribute to cardiac remodeling and contractile failure [43]. Here (P)RR gene transfer increased apoptotic cell death, and similar to prevention of HSP27 phosphorylation, apoptotic cell death was reduced by losartan in (P)RR-treated hearts. Collectively, these results suggest a specific role for Ang II in mediating the activation of signaling pathways and following cellular events (hypertrophy, contractile dysfunction, apoptosis, changes in extracellular matrix, angiogenesis) during myocardial remodeling process triggered by (P)RR (summarized in Figure 16). Moreover, the Ang II-independent alterations in extracellular matrix and contractile genes seem to be mainly responsible for worsening of cardiac function by (P)RR gene delivery.

PLZF acts as direct protein interaction partner of the (P)RR, and following the activation of (P)RR by renin, PLZF translocates to the nucleus and represses transcription of the (P)RR itself [36]. In our study (P)RR overexpression increased protein levels of PLZF and interaction between (P)RR and PLZF suggesting stronger repression of (P)RR transcription. Interestingly, this negative feedback mechanism was not significantly attenuated by losartan treatment. The importance of this finding remains to be studied further, as PLZF also interacts with AT_2_ receptor, mediating the antifibrotic and antiproliferative effects of Ang II [2].

Recent studies show that deletion of (P)RR results in the dysfunction of V-ATPase and implicate (P)RR also in the Wnt/β-catenin signaling [8]–[10]. Cruciat et al. [9] confirmed that (P)RR binds to the V-ATPase subunits, the accessary protein ATP6AP2 of V-ATPase being a truncated version of the (P)RR, as well as to Frizzled-8 and LRP-6. In the present study (P)RR gene transfer did not affect Wnt-3, β-catenin, Frizzled-8 and V-ATPase A1 protein levels in the left ventricle, thereby implicating that (P)RR-induced myocardial effects are not mediated via Wnt/β-catenin signaling pathway. However, these results do not exclude the possibility that overexpression of (P)RR could cause an imbalance in V-ATPase subunit assembly and therefore disruption of vacuolar acidification, leading to observed structural and functional changes. Interestingly, histological examination of the mice with cardiomyocyte-specific ablation of (P)RR, the gene product of *Atp6ap2*, revealed clusters of degenerating cardiomyocytes with extensive vacuolation, especially in the perinuclear region [10]. In contrast, in our present study, (P)RR gene transfer into left ventricle increased apoptotic cell death. These results suggest that the gene products of *Atp6ap2* may affect in two ways on cell survival: first as (P)RR, exerting Ang II-dependent apoptosis, and secondly, as the V-ATPase–associated protein, exerting non–RAS-related functions that are essential for cell survival.

Despite that the approach used in the present study effectively influences global cardiac function, it is known that it does not lead to global heart transduction, but instead results in marked expression throughout the LV free wall [12], [28], [29], [44]. For example, in the study of Szatkowski et al. [12] the entire LV free wall showed transgene expression with little or no detectable expression in remote areas (the right ventricle or interventricular septum), in agreement with the findings of our current study. Since the local LV injection of adenoviral constructs targets high expression of the transgene in the left ventricular free wall, the alterations in interventricular septum produced by (P)RR gene transfer might be a consequence of compensatory mechanisms for deteriorated LV function. Yet, the precise mechanisms contributing to changes in interventricular septum thickness by (P)RR gene delivery remain an important question for future studies.

In conclusion, neurohumoral blockers are key to pharmacological treatment of patients with impaired ventricular function and symptoms of heart failure [1]. Drugs that block RAS have substantially improved morbidity and mortality in these patients. Nevertheless, rates of morbidity and mortality are still high in patients with heart failure. Our studies indicate that local (P)RR gene delivery has deleterious effects on cardiac function. Moreover, our results provide the direct experimental *in vivo* evidence that (P)RR blockers may display additional cardiac effects on top of effective RAS blockade, because (P)RR activation induced distinct Ang II-independent extracellular matrix remodeling and worsening of cardiac function. (P)RR blockers may allow more complete myocardial protection in combination of effective AT_1_ receptor blockade and prevent the deleterious Ang II-independent actions of renin that are not inhibited by renin inhibitors. Interestingly, Batenburg et al. [45] showed recently that significant Ang II generation resulting from prorenin-(P)RR interaction occured at lower prorenin levels than direct ERK1/2 activation suggesting that signaling derived from (pro)renin-(P)RR interaction may be concentration-dependent.
